# Design and CFD Analysis of the Fluid Dynamic Sampling System of the “MicroMED” Optical Particle Counter †

**DOI:** 10.3390/s19225037

**Published:** 2019-11-19

**Authors:** Giuseppe Mongelluzzo, Francesca Esposito, Fabio Cozzolino, Gabriele Franzese, Alan Cosimo Ruggeri, Carmen Porto, Cesare Molfese, Diego Scaccabarozzi, Bortolino Saggin

**Affiliations:** 1 INAF—Astronomical Observatory of Capodimonte, Salita Moiariello 16, 80131 Naples, Italy; francesca.esposito@inaf.it (F.E.); fabio.cozzolino@inaf.it (F.C.); gabriele.franzese@inaf.it (G.F.); alan.ruggeri@inaf.it (A.C.R.); carmen.porto@inaf.it (C.P.); cesare.molfese@inaf.it (C.M.); 2 Department of Industrial Engineering, University of Naples “Federico II”, Piazzale Tecchio 80, 80125 Naples, Italy; 3 Department of Mechanical Engineering, Polo Territoriale di Lecco, Politecnico di Milano, Via Gaetano Previati 1c, 23900 Lecco, Italy; diego.scaccabarozzi@polimi.it (D.S.); bortolino.saggin@polimi.it (B.S.)

**Keywords:** MicroMED instrument, ExoMars 2020 mission, CFD, Mars

## Abstract

MicroMED is an optical particle counter that will be part of the ExoMars 2020 mission. Its goal is to provide the first ever in situ measurements of both size distribution and concentration of airborne Martian dust. The instrument samples Martian air, and it is based on an optical system that illuminates the sucked fluid by means of a collimated laser beam and detects embedded dust particles through their scattered light. By analyzing the scattered light profile, it is possible to obtain information about the dust grain size and speed. To do that, MicroMED’s fluid dynamic design should allow dust grains to cross the laser-illuminated sensing volume. The instrument’s Elegant Breadboard was previously developed and tested, and Computational Fluid Dynamic (CFD) analysis enabled determining its criticalities. The present work describes how the design criticalities were solved by means of a CFD simulation campaign. At the same time, it was possible to experimentally validate the results of the analysis. The updated design was then implemented to MicroMED’s Flight Model.

## 1. Introduction

**Figure 1:**
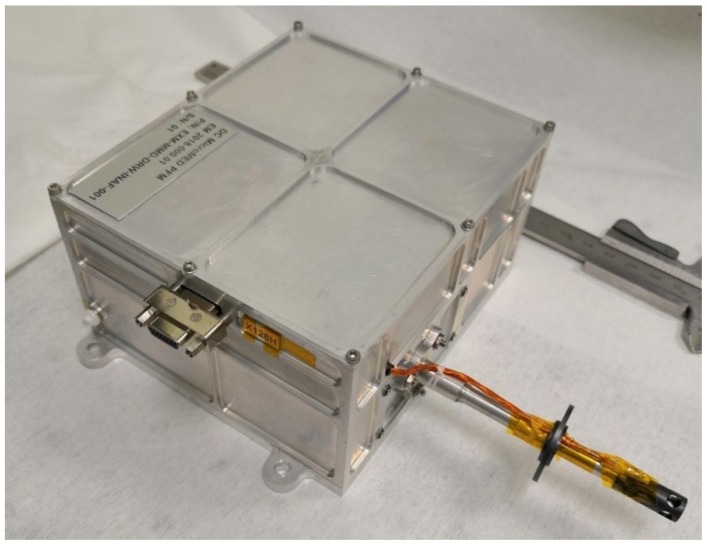
MicroMED’s proto Flight Model.

The optical particle counter MicroMED (Figure 1) [1,2,3,4,5] is conceived to provide the first ever in situ measurements of airborne dust in Martian atmosphere. The sensor will be part of the upcoming ExoMars 2020 mission, and it is a miniaturized version of the sensor MEDUSA [6,7], previously developed at the INAF (Istituto Nazionale di AstroFisica) Astronomical Observatory of Capodimonte (OAC) in Naples, Italy, where the characterization of dust in Earth and planetary atmospheres has been the main focus of the research activities for years [8,9,10,11,12,13,14]. MicroMED will be able to directly determine both the size distribution and concentration of dust grains suspended in Martian atmosphere (in the 0.4–20 µm diameter range), which is a measurement that has only been performed indirectly so far, using the light scattering characteristics of the aerosol [15]. Such measurement could have a huge impact on our understanding of Martian climate, on the mechanism of saltation and dust lifting on Mars, as well as phenomena such as dust devils and dust storms [16]. The size distribution of suspended dust is indeed a key input parameter for the mesoscale climatic models. Given that dust absorbs solar radiation, the size distribution alters the quantity of solar radiation that is able to reach the Martian surface, thus influencing the values of atmospheric temperature. Moreover, the dimension of suspended dust grains is directly related to the wind speed present on the surface, since the higher the wind speed, the larger the grains lifted [17].

The instrument analyzes dust by means of an optical system, including an optical collimating system, a laser diode emitting a laser beam and a light trap that avoids reflections and is able to detect the light scattered by the dust grains, a parabolic mirror that is able to focus the scattered light on a photodiode, and the instrument electronics that process the received input and characterize it. In order to allow the detection, the fluid with embedded particles has to cross a 1 mm^2^ sensing spot where the laser beam is focused. To do so, a proper fluid dynamic system was developed. Such a system is made of a sampling head that is exposed to outer atmosphere and able to collect dust grains, an inlet duct that conveys the fluid toward the sensing volume, an outlet duct that allows the expulsion of the sucked fluid after the optical sampling, and a pump that generates a pressure difference between the inlet and outlet sections of MicroMED, triggering the flow. While crossing the sensing volume, dust grains scatter light differently depending on their size and speed, so the amplitude and duration of the signals are directly related to those characteristics of the dust grains. The instrument’s Elegant Breadboard was realized, and tests showed that the instrument could not detect large dust grains (15–20 µm in diameter) with high efficiency, especially in the presence of wind [1,2]. These results highlighted the need for a detailed fluid dynamic analysis of the instrument design in order to solve its criticalities. In those studies, MicroMED’s Elegant Breadboard design was analyzed by means of the CFD code Fluent^®^, which allowed the identification of the causes of the observed low efficiency. Such causes were especially present for cold instrument conditions (the minimum operative temperature is around 253 K, given that MicroMED will be under a thermal cover limiting its temperature range to 253–313 K). Such studies also determined the optimum operating conditions of the pump needed to maximize MicroMED’s efficiency. The analysis highlighted the causes of the reduction of the instrument’s efficiency. A couple of undesired phenomena were individuated and will be described in the following sections. Then, the CFD analysis was enhanced in order to find a solution to such issues and improve the fluid dynamic design of MicroMED.

MicroMED’s design was updated to what is now the Flight Model design in order to correct those issues. The results for both CFD runs and laboratory tests show that a relevant improvement of the instrument’s efficiency has been obtained.

## 2. Methods

The analysis was performed by means of the version 18.1 of the CFD solver “Ansys Fluent^®^”. CFD numerical simulations are often used to study the movement of dust and aerosols in atmosphere [18,19,20]. As is well known, CFD methods are based on the conservation equations of mass, momentum, and energy for the flow (the equations are reported and discussed in Appendix A), which are valid only if the continuum hypothesis is valid. To verify that, the Knudsen number (Kn) for MicroMED was calculated (Kn is the ratio of the gas mean free path to the instrument characteristic length), and it was verified that Kn < 0.1 (the regime is considered continuum if Kn < 0.1, while in transitional regime if 0.1 < Kn < 50). For our applications, Kn was indeed 0.007, allowing the use of conventional Navier–Stokes based solvers such as Ansys Fluent.

The simulation campaign was performed following a logic similar to the previous analyses [1,2]. The analysis is focused on the main fluid dynamic parameters and on the “sampling efficiency” parameter, which is the ratio of the number of dust grains that cross the laser-illuminated spot to the total number of dust grains that cross the instrument’s inlet holes. This parameter allows evaluating the quality of the fluid dynamic design. Simulations considered different conditions in terms of ambient temperature and pressure in the range expected on Mars. Runs for five different values of ambient temperature between 190 and 280 K and for three different ambient pressure values (between 6 and 8 mbar) were performed. Seven different instrument temperatures inside the possible temperature range (253–313 K) were considered. Given that 95.3% of Mars’ atmosphere is made of carbon dioxide, runs were performed with CO_2_ as the fluid. The suction of fluid was simulated by means of the pressure difference generated by the pump between the inlet and outlet sections of MicroMED. For the Elegant Breadboard, the inlet–outlet Δp was simulated between 250 and 500 Pa. In the present work, simulations considered a Δp in the 100–300 Pa range, which is in accordance with the experimental results obtained by tests performed on the pump of MicroMED’s Flight Model. Most simulations were performed with a simple model not considering surface roughness. Simulations considering a mean surface roughness of 10–20 µm were indeed performed, and the results showed that the instrument’s sampling efficiency is barely influenced by surface roughness (variations always under 2% and most times under 1%). The analysis showed that the regime could be considered laminar similarly to what was obtained for the Elegant Breadboard design [1], allowing the use of laminar model for simulations. Indeed, the Reynolds number for the present application is always under 1000 given both the extremely low density of Mars atmosphere (1.6–1.8 × 10^−2^ kg/m^3^) and the small characteristic dimensions of MicroMED (order of magnitude of millimeters). This, coupled with a high Knudsen number related to the particles’ diameter (Kn_P_ ranges from 0.67 to 33.45), highlights the need for a correction factor in the drag law of the grains. In particular, the Cunningham correction factor for drag law was introduced [21]. The flow can be considered compressible similarly to what happened for previous works [1]. Dust grains in the sampling range have a Stokes number that ranges from 2 × 10^−4^ to 0.54, meaning that there could be a different behavior between large (15–20 µm) and small (0.4–1 µm) dust grains, with small dust grains more likely to follow the fluid streamlines along their entire path through the instrument. In CFD simulations, dust grains were simulated as spherical. Injections of dust grains of 16 different dimensions in the instrument’s sampling range were simultaneously simulated. The interaction among dust grains and the effect of magnetic and electrical forces on grains were calculated, and given the small effect on the overall results (the maximum contribution of such forces to dust grains speed is in the order of 10^−5^ m/s), they have not been considered during simulations. The CFD model was already validated in previous works [1], showing how the model prediction matched test results with good accuracy.

## 3. Undesired Phenomena

Previous CFD analyses showed a couple of criticalities in the Elegant Breadboard design, causing a reduction of the sampling efficiency for both large and small dust grains, with different extents and causes. These phenomena are described hereafter.

### 3.1. Collisions on the Inlet Walls

**Figure 2:**
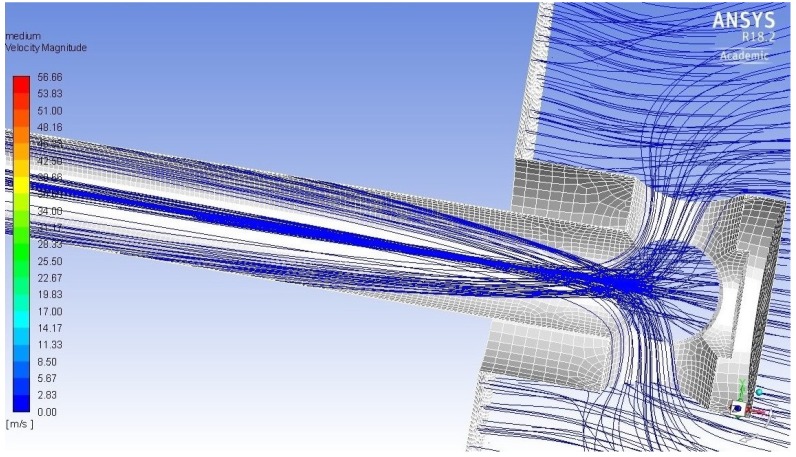
Particles trajectories inside the Elegant Breadboard’s inlet head and duct.

MicroMED’s sampling head is exposed to Martian atmosphere. A pump, connected to the outlet section of the instrument, generates a pressure difference with respect to the outside of MicroMED, triggering the suction of fluid. Then, an inlet duct conveys the fluid toward the sensing volume. The fluid drags the suspended particles, which follow the streamlines along the inlet duct. Given the particular geometry of the Elegant Breadboard’s inlet head, fluid streams coming from opposite holes of the sampling head tend to cross (see Figure 2). The sharpness of the bending depends on the dust grains inertia: the lower the inertia, the sharper the deflection. This is an important aspect, as large dust grains are a lot more likely to hit the duct walls. Given the aforementioned assumption that the walls’ mean surface roughness is of the same order of magnitude of the particles’ diameter (in the 10–20 µm range), this may cause adhesion of the particles to the walls, preventing their detection. The Elegant Breadboard’s duct shape promoted such phenomenon. Thus, the Flight Model design was modified to avoid the crossing of the trajectories and to linearize the fluid streamlines during the suction, also helping the laminarity of the flow, which is a design parameter (since it improves the instrument efficiency). In Figure 2, it is possible to see the dust grains’ trajectories inside the sampling head and inlet duct, showing the phenomenon just described.

### 3.2. Deflection of Dust Grains’ Trajectories

**Figure 3:**
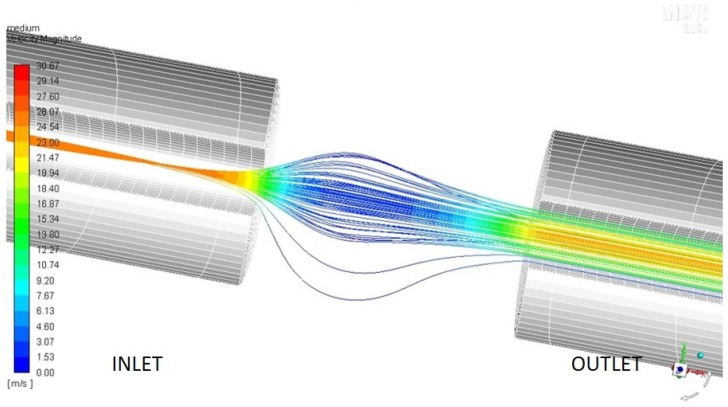
Dust grains behavior inside the sensing section of MicroMED’s Elegant Breadboard.

Inside MicroMED’s optical head and at the end of the inlet duct, a 4 mm gap is present, which is needed for the optical scan of the flow. The final section of the inlet duct has a 1 mm internal diameter. When the fluid reaches such gap, it expands, possibly deflecting the particles’ trajectories. There is indeed the chance that some of the dust grains follow the streamlines and cross the sensing plane outside the 1 mm^2^ laser illuminated spot, preventing their detection. This behavior is especially possible for small dust grains given their low Stokes number. Such phenomenon could alter the efficiency of MicroMED’s Elegant Breadboard depending on the environmental conditions. Therefore, the geometry update was aimed at having good performances in every possible operating condition. Figure 3 shows the possible undesired behavior of particles.

## 4. Geometry Update

**Figure 4:**
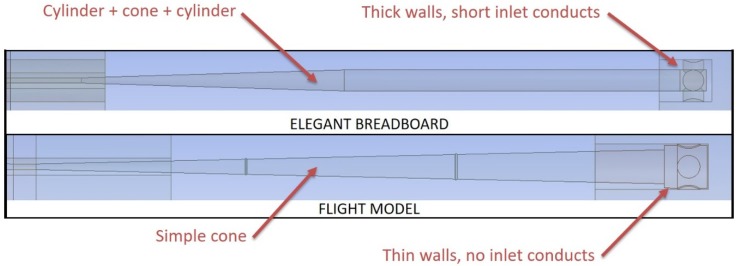
Geometry variations of MicroMED’s sampling head and inlet duct (drawings not to scale).

The fluid dynamic analysis of MicroMED’s Elegant Breadboard highlighted the need to change the shape of the inlet head. Then, the inlet walls’ thickness was reduced so that the small inlet cylindrical ducts that conveyed the fluid toward the main inlet duct disappeared. This variation helped reduce the curvature radius of the inhaled particles’ trajectories. The sampling head internal shape was also slightly modified and made more conical with respect to the mostly cylindrical shape of the Elegant Breadboard. This variation sharpened the particles deflection, allowing a less complex inlet duct. For the Flight Model, indeed, the inlet duct has a simply conical shape compared to the conical-then-cylindrical-then-conical shape present in the previous design (see Figure 4). These variations led to a big improvement of the duct capability to direct dust grains toward the sensing spot, as will be shown hereafter. Figure 4 shows the geometry variations adopted for the inlet head and duct.

**Figure 5:**
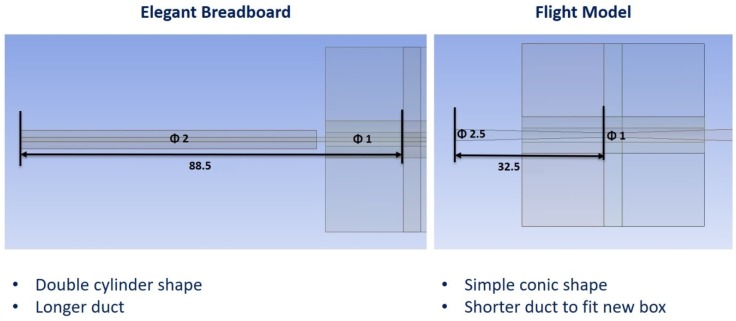
Geometry variations of MicroMED’s outlet duct (dimensions in mm).

The outlet duct of MicroMED also had to be changed because of volumetric constraints. The variations made the outlet more compact and short (32.5 mm against 88.5 mm of the previous version), and the internal duct was designed as simply conical instead of a combination of two cylindrical ducts. Figure 5 shows such variations. Section 5 will detail the effects of all these variations on MicroMED’s efficiency.

## 5. Results

Results are here reported as a comparison with the Elegant Breadboard’s status, showing the improvements obtained. The effect of environmental parameters on MicroMED’s efficiency was previously analyzed [1] for the Elegant Breadboard. The present analysis shows similar effects for the Flight Model. The instrument temperature is the most influential parameter both on the sampling efficiency and in the evaluation of the volumetric flow rate, which is needed in order to determine the dust concentration in the sample of gas inhaled. The analysis performed on the Elegant Breadboard [1] showed that other parameters can influence MicroMED’s behavior. Moreover, the optimum conditions for tests had to be deduced, since good efficiency was not guaranteed in any environmental conditions. The Flight Model design provides improvements of the efficiency for every size and basically guarantees good efficiency for every possible environmental condition. The analysis performed on the Flight Model also shows how optimum results can be obtained with a Δp generated by the pump in the 200–300 Pa range. The Elegant Breadboard needed at least 300 Pa Δp in order to work, so the current design allows more flexibility in the choice of the operating conditions and a reduction of the power consumption of the instrument. The following sections describe the results obtained for such an updated design.

### 5.1. Sampling Efficiency of Large Dust Grains

**Figure 6:**
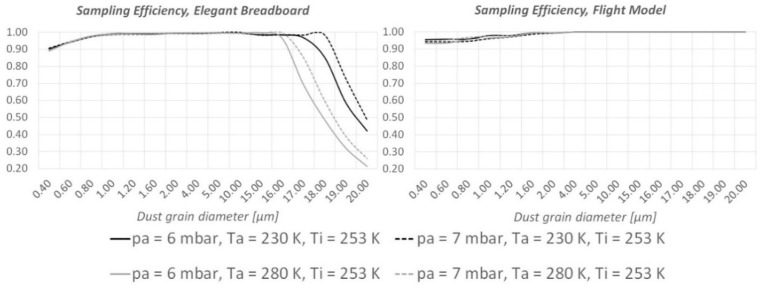
Comparison between the sampling efficiencies of MicroMED’s Elegant Breadboard and Flight Model for “cold instrument conditions” (T_i_ = 253 K).

**Figure 7:**
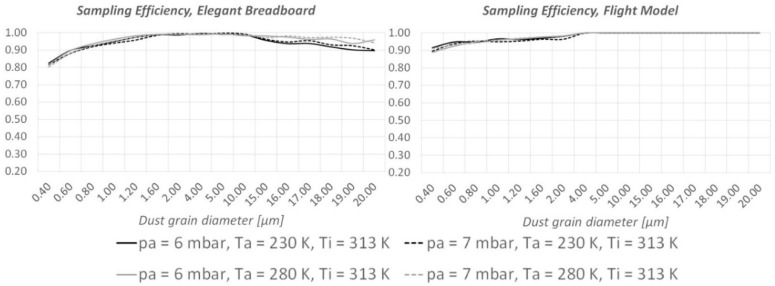
Comparison between the sampling efficiencies of MicroMED’s Elegant Breadboard and Flight Model for “hot instrument conditions” (T_i_ = 313 K).

As shown in Figure 6 and Figure 7, CFD runs predict an important improvement of the instrument’s ability to detect large (15–20 µm diameter) dust grains. When the instrument is “hot” (T_i_ = 313 K, the maximum allowed temperature under the thermal cover), MicroMED’s Breadboard was already sufficiently efficient for such dust sizes; however, the updated design provides improvements as large as 10–14%. When the instrument is “cold” (T_i_ = 253 K, the minimum temperature allowed), the improvement is clear. The impacts of dust grains on the walls completely disappear, so that 100% of the large dust grains can be correctly detected by MicroMED’s optical system compared to roughly 30% obtained with the Elegant Breadboard design (see Figure 6).

**Figure 8:**
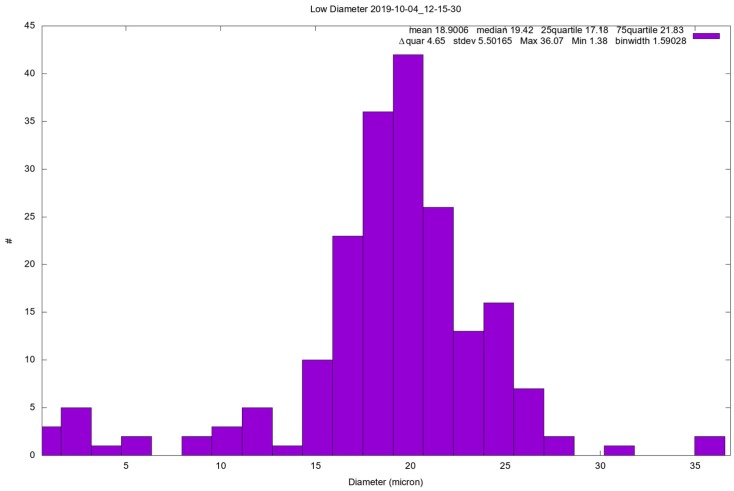
Example of test performed at the Capodimonte laboratory injecting 19.7-µm calibrated SiO_2_ particles.

The instrument’s ability to detect large dust grains was confirmed by tests performed at the INAF Astronomical Observatory of Capodimonte Laboratory. In such a laboratory, a Martian chamber and a clean room are installed, enabling the reproduction of Martian conditions in terms of pressure and atmospheric composition, thus reducing the amount of atmospheric dust that could alter the measurements and keep the instrument sterile in accordance to the planetary protection constraints. Moreover, the ATS (Autonomous Thermal Simulator) system installed in the Capodimonte laboratory allowed to perform tests at different instrument temperatures, simulating the possible different conditions foreseen at the lander level during the mission. During such tests, the instrument appeared to show a good ability to detect large dust grains, as Figure 8 shows, even though the results of the analysis are still preliminary, so they are only mentioned. The test showed in Figure 8 was made injecting in the Martian chamber monodispersed SiO_2_ 19.7 µm spherical calibrated particles (for the test setup, see Appendix B).

### 5.2. Dust Grains’ Position in the Sensing Plane and Evaluation of Dust Grain Size Distribution

**Figure 9:**
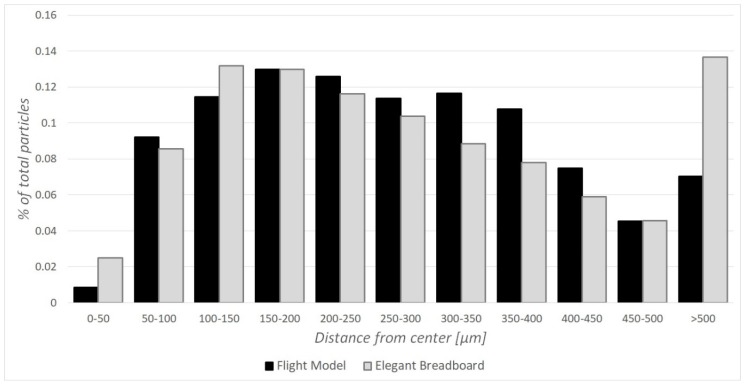
Example of position histogram comparing the distance of crossing particles from the center of the laser spot for both the Elegant Breadboard (GRAY) and the Flight Model (BLACK) design.

Particles trajectories along MicroMED were analyzed in order to understand not only if they cross the laser-illuminated spot, but also how far from the center of the spot they do so. Indeed, given that laser light is more uniform and intense at the center of the laser spot, errors in the determination of the dust grain size are smaller when particles cross the spot close to the center. For this reason, position histograms were derived from CFD simulations, determining the quality of the fluid dynamic design. The Elegant Breadboard already guaranteed good results in such an aspect, and the geometry variations adopted for the new design could potentially make the particles more “spread” through the sensing plane. Indeed, in some cases, the streamlines’ deflection is smoother for the new design; therefore, the dust grains are more distributed inside the sampling spot rather than more concentrated. The comparison was made for two different working conditions, given that the optimum operating condition for the Elegant Breadboard version is related to an inlet–outlet pressure difference of 300 Pa while the Flight Model already works efficiently at 200 Pa, which was the operating condition considered in this paper. The results show that the new design does not alter the chances of a grain crossing the sampling spot in the proximity of the center (roughly 91% of all dust grains inhaled are within 400 µm, and 86% are within 350 µm from the center of the spot; these numbers pretty close to the ones obtained for the Breadboard design, see Figure 9). There are some cases where the efficiency for small dust grains could slightly decrease (by less than 3%) because of the dynamics previously described, but the geometry variation provides a definite improvement of the overall percentage of particles that are now detectable. For large dust grains (see Figure 6), there is a definite improvement (sampling efficiency 70% higher in some cases, as already stated). Moreover, the new design provides efficiency over 89% for all the small grains (diameter < 1 µm) for every possible environmental condition, differently to the Elegant Breadboard that had cases of efficiency dropping below 80%.

**Figure 10:**
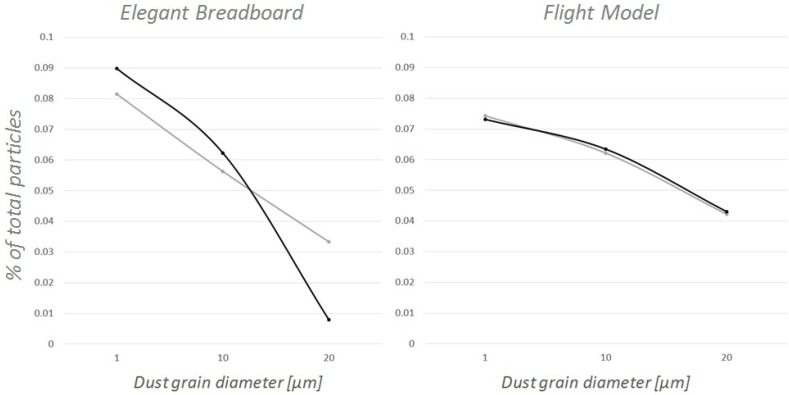
Comparison between the Elegant Breadboard’s and the Flight Model’s ability to correctly deduce size distribution (GRAY: real distribution, BLACK: measured distribution).

The new design also gave tangible improvements in terms of the instrument’s ability to evaluate dust grain concentration. Figure 10 shows that the Flight Model is able to describe the size distribution of the particles inhaled with much better accuracy with respect to the previous design.

### 5.3. Results in Presence of Wind

A CFD analysis to predict MicroMED’s behavior in windy environments was also performed, given that sustained wind is present in most occurrences on Mars. A CFD model was developed to simulate windy conditions, changing boundary conditions from the traditional CFD model used in this paper in order to generate a wind (of set speed) that passes over MicroMED’s sampling head. Dust grains could only be simulated as spherical, which is expected to make simulated particles more stable than real particles, so the results obtained could underestimate the instrument’s ability to detect particles while in the presence of wind (more stable dust grains are less probable to be deflected inside MicroMED). Indeed, while CFD analysis of the Elegant Breadboard stated that MicroMED could be unable to detect dust grains starting from a wind speed of 2 m/s, a test campaign performed at the Aarhus Wind Tunnel Simulator (AWTS) facility at Aarhus University in Aarhus, Denmark [22] showed that small dust grains are well detected, while the detection of large dust grains is related to a threshold value. For every size, there is a threshold value of wind speed after which MicroMED is unable to detect grains of such size: the larger the size of dust grains, the smaller the speed threshold value. The preliminary results of such a test campaign seem to show that the instrument was able to see dust grains for wind speeds up to around 10 m/s (see Appendix C for the description of test setup at the AWTS facility).

**Figure 11:**
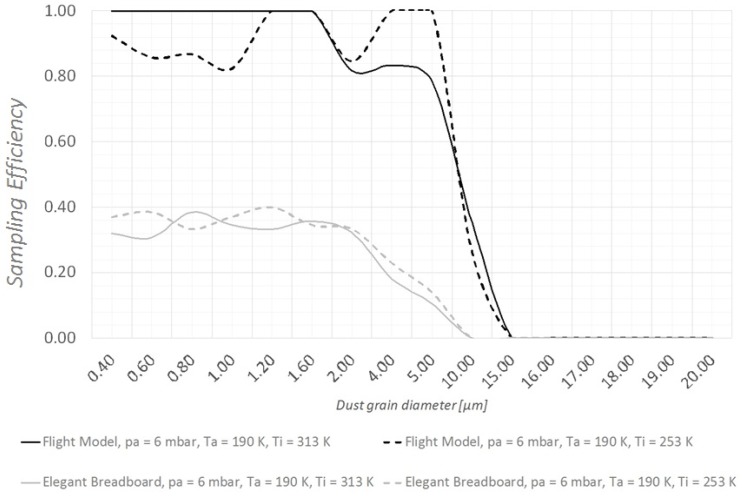
Comparison between Flight Model and Elegant Breadboard in terms of sampling efficiency, wind speed 2 m/s.

**Figure 12:**
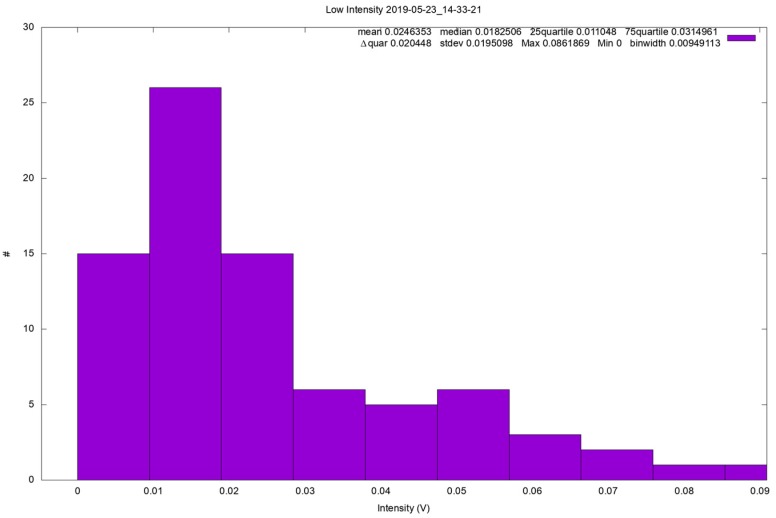
Example of test performed at AWTS in Aarhus, Denmark. The plot is reported in terms of signal intensity as the analysis is not ready; however, tests were performed with 20.07-µm particles only.

The results of CFD simulations on the Flight Model’s geometry, compared with those on the Elegant Breadboard, showed clear improvements in the ability to detect small dust grains and a moderate improvement in the ability to detect larger grains. Figure 11 shows a comparison between the two analyzed geometries, highlighting the better overall efficiency obtained. However, according to CFD analysis, efficiency is still low. Tests at the AWTS were also performed on MicroMED’s Flight Model, and the preliminary results show that the improvement was probably bigger than what was predicted by the CFD. It was found that MicroMED’s updated design not only improves the instrument’s efficiency in the detection of large dust grains, but it also provides good efficiency. As Figure 12 shows, MicroMED was able to detect 20-µm diameter particles even at the highest possible wind speed for the facility (15 m/s), which confirms that the CFD model is conservative, and that MicroMED’s Flight Model better detects dust grains also in the presence of wind. Since the analysis of the measured signals is still ongoing, the figure reports data in terms of signal intensity (as measured by the instrument’s detector) and not in size; however, the run relative to such a figure was performed injecting only 20.07-µm calibrated spherical particles with a wind speed of 15 m/s, so the signals detected are for sure 20.07-µm dust grains. The figure was still reported to show that hundreds of samples can be obtained in such tests. Such results confirm that the CFD model should be improved to predict MicroMED’s behavior in windy conditions with better accuracy. Moreover, the Flight Model’s ability to work properly with lower pump rpm speeds could help, as it could be possible to increase the pump speed if necessary. However, the analysis of data obtained in such a test campaign (plenty of tests were performed with 10 different monodispersed spherical sizes and with JSC-1 non-spherical Martian simulant, as well as tests with other broad distribution samples) is still preliminary; therefore, they are only mentioned in this work.

## 6. Conclusions

MicroMED’s Flight Model design was developed by means of a CFD simulation campaign aimed at the improvement of the instrument’s ability to detect dust grains, especially large ones (15–20 µm in diameter), in every possible environmental condition that the instrument could face during the ExoMars 2020 mission. This is important since the actual operating conditions of the instrument while on Mars are unpredictable. The analysis shows that the updated fluid dynamic design improves the detection of dust grains. The key result is a huge improvement in the ability to suck and detect large dust grains, avoiding hits on the walls and obtaining more accurate results in the measurement of size distribution curves for the samples. Optimum operating conditions can be obtained for an extended range of pump-generated Δp, which allows more flexibility in the choice of operating conditions in relation to the environment. The analysis results were confirmed by tests performed at the Astronomical Observatory’s Laboratory in Naples, Italy, where abundant samples of large dust grains were detected by the instrument. The analysis was also extended to windy operating conditions. The model showed to be extremely conservative in predicting the outcome of tests. However, CFD results predicted an efficiency improvement, which was confirmed by tests performed at the AWTS facility in Aarhus, Denmark. Indeed, tests showed that MicroMED’s Flight Model is able to detect significant amounts of large dust grains even at high wind speed (speeds until 15 m/s were tested), confirming that an improvement was obtained, as predicted by the CFD model.

## Appendix A

This appendix shows the equations used by ANSYS Fluent to solve the flow for the present application. Given that laminar flow is present, conservation equations of mass and momentum are reported for such a regime. The equations are the following:

$$\frac{\partial \rho }{\partial t}+\nabla \cdot \left(\rho \vec{v}\right)=S_{m}$$

$$\frac{\partial \left(\rho \vec{v}\right)}{\partial t}+\nabla \cdot \left(\rho \vec{v}\vec{v}\right)=-\nabla p+\nabla \cdot \left(\overset{=}{\tau }\right)+\rho \vec{g}+\vec{F}$$

where ρ is density, $\vec{v}$ is the fluid velocity vector, *S_m_* is the mass added to the continuous phase from the dispersed second phase (dust), *p* is the static pressure, $\overset{=}{\tau }$ is the stress tensor, and $\rho \vec{g}$ and $\vec{F}$ are the gravitational body force and the external body force (e.g., that arise from interaction with the dispersed phase). Such equations are valid also for compressible flows. An energy equation has to be added because thermal effects are also considered in simulations. The energy equation is

$$\frac{\partial \left(\rho E\right)}{\partial t}+\nabla \cdot \left(\vec{v}\left(\rho E+p\right)\right)=\nabla \cdot \left(k_{eff}\nabla T-\underset{j}{\sum }h_{j}{\vec{J}}_{j}+\left({\overset{=}{\tau }}_{eff}\cdot \vec{v}\right)\right)+S_{h}$$

where *k_eff_* is the effective conductivity (*k_eff_* = *k* + *k_t_*, where *k_t_* is the turbulent thermal conductivity that is not active for the laminar model) and, ${\vec{J}}_{j}$ is the diffusion flux of species (no flux here). The first three terms of the right-hand side of the equation represent energy transfer due to conduction, species diffusion, and viscous dissipation, respectively. *S_h_* includes effects due to chemical reaction and other contributions that are not present in this work. In these equations, the stress tensor $\overset{=}{\tau }$ is expressed as:

$$\overset{=}{\tau }=\mu \left[\left(\nabla \vec{v}+\nabla {\vec{v}}^{T}\right)-\frac{2}{3}\nabla \cdot \vec{v}I\right]$$

where *μ* is molecular viscosity, *I* is the unit tensor, and the second term on the right-hand side is the effect of volume dilation.

## Appendix B

This appendix describes the procedure used for tests performed at the INAF–OAC laboratories of Capodimonte in Naples, Italy.

Tests were performed inside the INAF–OAC clean room, which includes a vacuum chamber. All the components inside the clean room have to be sterilized to avoid contamination of the instrument in accordance with the planetary protection constraints.

The tests simulated the data acquisition of MicroMED under Martian conditions. For this reason, an atmosphere with a pressure between 6 and 7 mbar was created inside the vacuum chamber.

Temperature conditions were set and controlled using the Autonomous Thermal Simulator (ATS) system, which is a custom thermal system produced by TransTech^®^ that allowed varying the instrument temperature in the 253–313 K temperature range.

The electrical and grounding setup can be accessed upon request to the authors of the present paper.

Tests were performed with calibrated SiO_2_ particles made by Microparticles^®^ of different diameters (1, 2, 4, 6, 8, 10, 15, 16, 18, and 20 µm) and with a JSC-1 Martian simulant (which is made of non-spherical particles).

As for the injection of particles, a Medikron^®^ atomizer containing a weighted sample of grains was used. The atomizer was filled with ethanol, mounted on a pulsing vortex mixer (to prevent the agglomerating of particles), and connected with a CO_2_ tank by means of a tube, so that the injection of CO_2_ allowed the exit from the atomizer of a mushroom cloud of ethanol and dust. Ethanol tends to evaporate because of the low pressure, leaving only silica particles available to MicroMED for suction.

Papers describing the experiments in detail and giving an in depth analysis of the results are forthcoming.

## Appendix C

This appendix describes the procedure used for tests performed at the AWTS facility at the University of Aarhus situated in Aarhus, Denmark. The test campaign was funded by the Europlanet 2020 project and allowed one week of detailed testing of the instrument under various conditions.

Tests were performed inside the facility’s vacuum chamber. The chamber allowed reproducing Martian conditions in terms of pressure, atmospheric composition, and wind speed. Indeed, a fan was present inside the vacuum chamber, allowing wind speeds varying from 0.5 to 15 m/s.

Temperature conditions were controlled by means of a base plate.

Electrical and grounding setup can be accessed upon request to the authors of the present paper.

The injection of particles was made by means of a valve. First, 1 mg of grains (monodispersed calibrated SiO_2_ particles made by Microparticles^®^ or a broad distribution sample of non-spherical grains depending on the test) was positioned in a small duct connected to the valve. The opening of the valve caused suction of the dust grains because of the pressure difference between the inside and the outside of the chamber (6–8 mbar inside, 1 bar outside). Then, the flow generated by the fan moved the particles toward MicroMED, reproducing the dust-embedded wind expected on Mars.

Tests were performed with calibrated, spherical particles of different diameters (1, 2, 4, 6, 8, 10, 15, 16, 18, and 20 µm), with a JSC-1 Martian simulant (which is made of non-spherical particles) and with other samples of broad distribution. Papers describing in detail the results of the test campaign are currently under development.
